# Freeze-Dependent Physiological and Transcriptional Changes in *Olea europaea* L. Cultivars with Different Cold Resistances

**DOI:** 10.3390/ijms26093934

**Published:** 2025-04-22

**Authors:** Maria Gladysheva-Azgari, Natalia Slobodova, Fedor Sharko, Artem Fatkulin, Svetlana Tsygankova, Valentina Tsiupka, Oksana Grebennikova, Iliya Bulavin, Eugenia Boulygina, Sergei Tsiupka

**Affiliations:** 1 National Research Center “Kurchatov Institute”, 123182 Moscow, Russiaeugenia.bulygina@gmail.com (E.B.); 2 N.V. Tsitsin Main Botanical Garden, Russian Academy of Sciences, 127276 Moscow, Russia; 3 Faculty of Biology and Biotechnology, HSE University, 101000 Moscow, Russia; aafatkulin@edu.hse.ru; 4 Nikita Botanical Gardens–National Scientific Centre of the Russian Academy of Sciences, 298648 Yalta, Russia

**Keywords:** cold resistance in plants, transcriptional changes in plants, freezing in plants, olive trees

## Abstract

Understanding the transcriptional responses of plants under cold stress conditions is critical for olive cultivation, particularly in regions prone to extreme weather fluctuations and especially with increasing threats from climate change. In controlled experiments, we subjected leaves of three cold-tolerant and three cold-susceptible cultivars to moderate (−7 °C) and severe (−12 °C) freezing stress, followed by recovery at baseline temperatures. The study measured photosynthetic efficiency and enzymatic activity and showed physiological and gene expression changes using different methods. Distinct transcriptomic adaptations were revealed. Cultivars displayed enhanced differential expression associated with photosynthetic recovery and gene regulation in metabolic pathways. Two overlapping DEGs with increased expression were found in all cultivars during initial freezing.

## 1. Introduction

The olive tree (*Olea europaea* L.) holds immense agronomic and economic significance, particularly in the Mediterranean region and adjacent areas, where it has been cultivated for millennia. However, the increasing frequency and intensity of weather fluctuations due to climate change pose substantial challenges for olive cultivation [1]. Cold stress, in particular, is highly detrimental, affecting the growth, productivity, and survival of olive trees [2,3].

The plant response to cold is a multifaceted physiological process. During acclimation, resistance to low temperatures increases through various metabolic and structural adaptations, especially in the leaves, which are the primary sites of photosynthesis. Cold stress can disrupt cellular homeostasis, leading to oxidative stress, membrane damage, and reduced photosynthetic efficiency [4]. Cold-tolerant olive cultivars often exhibit enhanced defense mechanisms, such as the accumulation of osmoprotectants and antioxidants and the stabilization of cellular structures [5].

Low-temperature stress manifests at all levels of the plant organism. At the cellular level, frost damage primarily affects membranes [6,7], leading to an increase in reactive oxygen species (ROS), which can subsequently cause lipid peroxidation, membrane degradation, protein breakdown, and metabolic disruption [8]. Mechanisms for the removal of ROS have been developed, utilizing both enzymatic and non-enzymatic antioxidant systems. These include redox enzymes (superoxide dismutase, catalase, peroxidase, etc.) and protective compounds (phenolic substances, ascorbic acid, proline, etc.) to mitigate or protect against oxidative damage caused by subzero temperatures in plants [4,9].

Among low-molecular-weight compounds, phenolic substances play a significant role in the formation of olive tree resistance to low temperatures. The influence of phenolic compound content on the cryoresistance of olive cultivars has been described in numerous studies [7,10,11,12].

Transcriptomic changes play a central role in cold adaptation and response. Exposure to low temperatures triggers a cascade of gene expression changes aimed at reducing cold-induced damage. Key genes involved in stress signaling, transcription factors, cryoprotective proteins, and metabolic adjustments show differential expression during cold stress [13]. Previous studies have provided insights into these mechanisms in plants generally and in olives specifically. For example, it has been shown that most molecular pathways involved in cold response in olives align with those in other plants [13], including the induction of genes coding for modifications in membrane lipid composition, ROS scavenging systems, CBF family genes, and dehydrins, the suppression of photosynthesis-related genes, and the activation of carbohydrate catabolism. While cold responses within a single olive cultivar conform to expected outcomes, the mechanisms underlying these responses are believed to differ between cultivars with different cold tolerances.

Geographical differences also significantly influence the cold response of olive cultivars. For instance, cultivars grown in Italy may exhibit different cold tolerance profiles compared with those grown in Crimea due to variations in local climates, soil conditions, and historical cultivation practices. Research indicates that the same olive cultivar can be classified as cold tolerant or non-tolerant depending on the growing conditions, highlighting the critical role of local adaptation and microclimatic factors in determining the categorization of a cultivar. For example, cv. ‘Leccino’ is considered a cold-tolerant cultivar in the foreign literature, while on the southern coast of Crimea it is regarded as non-tolerant. Studies by Azzarello et al. [14], based on electrolyte leakage, impedance spectroscopy, and fractal analysis, classified cv. ‘Leccino’ among plants with high frost resistance. Similar results were reported by Alfei et al. [15] on plants exposed to natural freezing conditions. However, according to V.P. Alekseev [16], the frost resistance of cv. ‘Leccino’ is described as satisfactory, while N.F. Sokolova [17] characterized it as having weak frost resistance. Low frost resistance was also noted by I.A. Zhigarevich [18], Gubanova et al. [19], and Tsiupka et al. [20].

Understanding the genetic mechanisms and transcriptomic responses underlying freezing stress in *O. europaea* L. cultivars is therefore of extreme importance.

The Nikitsky Botanical Garden (NBG RAS, Crimea), located on the southern coast of Crimea, maintains an extensive collection of olive cultivars [21]. The collection includes well-known cultivars commonly grown in the Mediterranean region as well as locally bred cultivars that exhibit cold tolerance under the conditions of the southern coast of Crimea.

The Crimean Peninsula represents one of the northernmost regions for olive cultivation, where winter temperatures can drop as low as −14.6 °C (Table 1) [22].

The presented climatic data for the 30-year period from 1991 to 2020 show a consistent warming trend, objectively characterizing the current climatic specifics of the studied region, and can be considered the modern climatic norm for meteorological parameters on the southern coast of Crimea [22]. The average of the absolute minimum temperatures over the last 30 years was −7.1 °C, and the absolute minimum temperature reached −12.4 °C. This supports the selection of −7 °C and −12 °C as experimental temperatures.

The average temperatures of the coldest months (January and February) during the period 1991–2020 were +3.7 °C and +3.6 °C, respectively, justifying the use of +4 °C as the control temperature (as the most probable temperature at which plant activity occurs during winter on the southern coast of Crimea).

As noted by several authors, when the temperature drops below −7 °C, olive trees may suffer leaf loss and the desiccation of young shoots, depending on the duration and severity of the cold, leading to significant yield losses. If the temperature reaches −12 °C, plants may die [23,24,25,26]. There are several distinctive characteristics of the cold response that attract researchers’ attention. The literature differentiates between chilling and freezing stress [27], where chilling stress refers to temperatures between 0 °C and 15 °C and freezing stress is described as any exposure to temperatures below 0 °C. However, different plants (including various olive cultivars) exhibit distinct responses to the range of freezing temperatures, further subdividing freezing stress into mild and severe stress. Severe leaf damage in olives is thought to occur at −7 °C [28], while the LT_50_ (the temperature at which half the plants die due to freezing injury) for olive leaves is approximately −12 °C [23]. It is also known that the LT_50_ and another important parameter for assessing freezing tolerance, the ice nucleation temperature (INT), vary among olive cultivars [29]. Not only is the initial response to cold important, but also the plant’s ability to recover quickly from exposure. For example, studies on cv. ‘Picual’ have shown that olives can fully recover within five days [30], but comparative analyses to determine recovery rates in cultivars with varying degrees of cold tolerance have not yet been conducted.

The aim of this study was to analyze the transcriptomic profiles of leaves from six *O. europaea* L. cultivars, including three cold-tolerant and three cold-susceptible ones, under controlled cold stress conditions. By subjecting the leaves of these cultivars to moderate (−7 °C) and extreme (−12 °C) freezing stress, we aimed to uncover the differential gene expression patterns and molecular pathways activated in response to varying degrees of cold stress.

## 2. Results

### 2.1. Phenotypic Changes

Under control conditions, the leaves of all studied *O. europaea* (L.) cultivars exhibited green to dark green coloration on the adaxial surface. No significant changes were observed after cold exposure at −7 °C for 12 h in either cold-tolerant or non-tolerant genotypes.

At −12 °C, chlorosis and necrosis were visible on the adaxial side of the leaf blade in the interveinal spaces. In cases of more severe damage, complete darkening of the leaf surface was observed, likely due to the oxidation of phenolic compounds that enter the intercellular space when the cell membrane ruptures. Leaf tissue death was clearly indicated by leaf curling and rapid desiccation.

**Figure 1:**
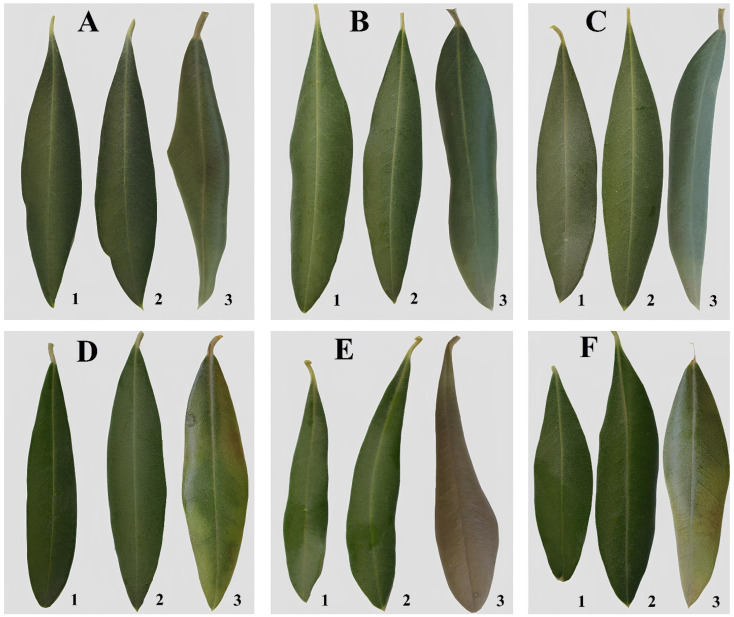
Visual assessment of leaf tissue damage following the freezing experiment. (**A**) cv. ‘Nikitskaya-2’, (**B**) cv. ‘Tiflisskaya’, (**C**) cv. ‘Tossiyskaya’, (**D**) cv. ‘Coreggiolo’, (**E**) cv. ‘Razzo’, (**F**) cv. ‘Leccino’. 1—Control, 2—Leaves after exposure to −7 °C, 3—Leaves after exposure to −12 °C.

The highest level of leaf cryoresistance was observed in the following cultivars: ‘Nikitskaya-2’, ‘Tiflisskaya’, and ‘Tossiyskaya’, whereas in the cultivars ‘Coreggiolo’, ‘Leccino’, and ‘Razzo’, chlorosis was prominently displayed, followed by complete desiccation of the leaf blade accompanied by darkening of the adaxial surface (Figure 1).

### 2.2. Structural Analysis of Cold-Susceptible and Cold-Tolerant Cultivars

**Figure 2:**
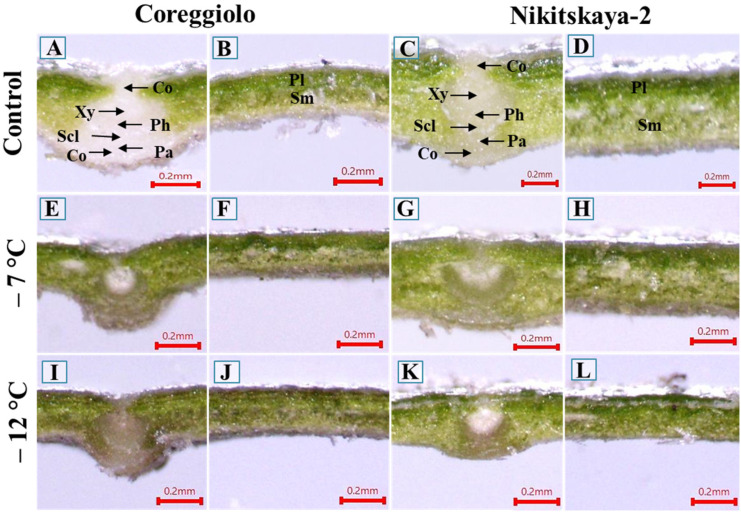
Leaf cross-sections after freezing temperature experiments. Co, collenchyma; Pa, parenchyma; Ph, phloem; Pl, palisade; Scl, sclerenchyma; Sm, spongy mesophyll; Xy, xylem (light microscopy).

The state of the leaf blade tissues of cv. ‘Coreggiolo’ and cv. ‘Nikitskaya-2’ under freezing temperatures was investigated. In the control, the epidermis, collenchyma, parenchyma, xylem, phloem, and sclerenchyma of the central vein were white or ashy colored. The palisade was yellowish-green and the spongy mesophyll had a fawn color (Figure 2A–D). At −7 °C, the epidermis, phloem, parenchyma, and collenchyma changed from white or ashen to a pale grayish color (Figure 2E–H). A temperature decrease to −12 °C provoked necrosis and the tissues became a chestnut-brown color (Figure 2I–L). Leaf lamina tissues acquired glassiness.

**Figure 3:**
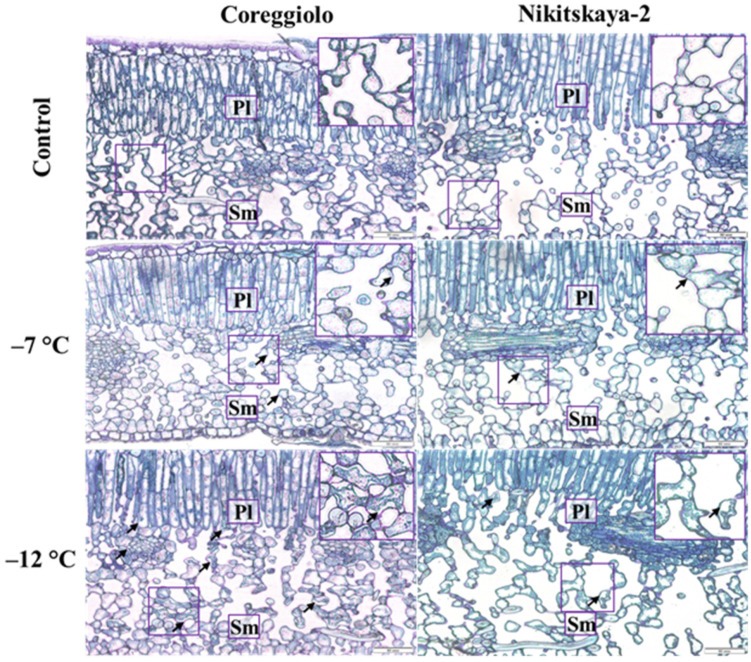
Leaf cross-sections after freezing temperature experiments. Pl, palisade; Sm, spongy mesophyll; Xy, xylem (light microscopy, toluidine blue staining). Arrows indicate sites of plasmalemma detachment.

At the tissue and cellular level, isolated incidents of plasmalemma detachment from cell walls were observed in the spongy mesophyll at −7 °C (Figure 3).

**Figure 4:**
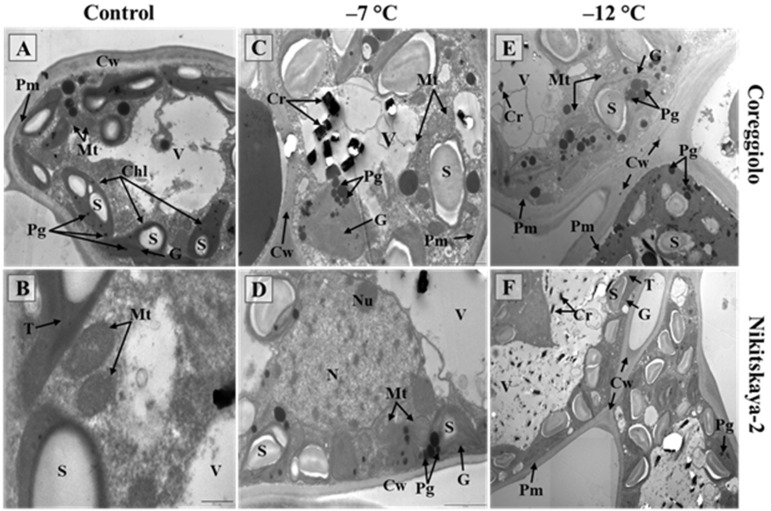
Spongy mesophyll ultrastructure after freezing temperature experiments. Cr, crystal; Cw, cell wall; G, granum; Pg, plastoglobulus; Pm, plasmalemma; Mt, mitochondrion; N, nucleus; Nu, nucleolus; S, starch grain; T, thylakoid; V, vacuole (transmission electron microscopy).

At −12 °C, changes in the spongy mesophyll occurred in groups of cells; however, the damage was stronger for cv. ‘Coreggiolo’ (Table 2). Cell ultrastructure analysis revealed chloroplast reactivity in the first instance. They became swollen, the inner membrane system gradually disappeared, and the plastoglobuli size and quantity increased (Figure 4C–D). At −12 °C, the freezing effect was more clearly observed (Figure 4E–F). In some cells with plasmalemma desquamation, organelles were difficult to distinguish in the hyaloplasm. It should be noted that the cell structure was preserved better in the leaves of cv. ‘Nikitskaya-2’.

### 2.3. Photosynthetic Apparatus

#### 2.3.1. Moderate Stress (−7 °C)

**Figure 5:**
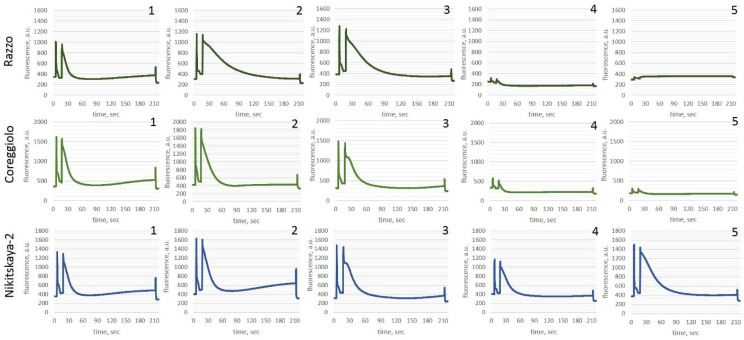
Changes in the chlorophyll fluorescence induction curve at various stages of cold stress (Kautsky curves). 1—Control (Point A), 2—after exposure to −7 °C for 12 h (Point C), 3—after 24 h of recovery following exposure to −7 °C (Point E), 4—after exposure to −12 °C for 12 h (Point C), 5—after 24 h of recovery following exposure to −12 °C (Point E).

Complete results are provided in Supplementary File S1, while the Kautsky curves are shown in Figure 5. Under normal functioning of the photosynthetic apparatus (control conditions, Point A), no significant differences were observed between cold-tolerant and non-cold-tolerant cultivars. At a temperature decrease to −7 °C (Point B), the first reaction to stress is the limitation of assimilation processes. The metabolic pathways activated by olive plants under these conditions are highly energy intensive and typically shift overall metabolism toward defense responses, thereby restricting all productive processes of photosynthesis.

In all studied olive cultivars, as the stress increased (Points B and C), a reduction in the effective photochemical quantum yield of Photosystem II (Y(II)) was observed. Energy previously allocated to photosynthesis was redistributed toward regulatory quenching mechanisms (Y(NPQ)) and thermal dissipation (Y(NO)). After the stress subsided (Points D and E), the plants restored photosynthetic activity to control levels, indicating no damage to the chlorophyll–protein complexes.

This temperature can thus be characterized as moderate stress, which does not cause significant cellular damage but temporarily alters plant metabolism and restricts the productive processes of photosynthesis.

#### 2.3.2. Severe Stress (−12 °C)

Under control conditions (Point A), the assimilation reflected the absence of stress, as indicated by the maximum photochemical quantum yield of PSII (Fv/Fm), which ranged from 0.52 to 0.70 relative fluorescence units. At the initial stage of stress exposure (Point B), all cultivars exhibited a decrease in photosynthetic activity. The strongest initial stress response was observed in the cultivars ‘Coreggiolo’, ‘Razzo’, and ‘Tiflisskaya’. At this stage, plants showed disruptions in physiological and biochemical processes along with the activation of defense responses to stress. The increasing role of adaptive mechanisms led to substantial metabolic costs and a decrease in photosynthetic productivity. All cultivars experienced a significant reduction in the effective photochemical quantum yield of Photosystem II (Y(II)) and an increase in regulatory mechanisms (Y(NPQ)).

Prolonged exposure to low subzero temperatures revealed threshold values distinguishing tolerant and non-tolerant cultivars. For example, after 12 h of exposure (Point C), cv. ‘Razzo’ showed a decrease in viability to sublethal levels. Most cultivars continued to exhibit decreases in photosynthetic activity, with suspected non-tolerant cultivars in particular showing an increase in non-regulated dissipation (Y(NO)), indicating irreversible damage to chlorophyll–protein complexes.

When the stress factor ceased and temperatures normalized (Point D), the cold-tolerant cultivars ‘Tiflisskaya’, ‘Tossiyskaya’, and ‘Nikitskaya-2’ activated repair processes that restored photosynthetic functions to normal levels. In contrast, the susceptible cultivars ‘Coreggiolo’, ‘Leccino’, and ‘Razzo’ showed suppressed physiological and biochemical processes.

The selected exposure duration (12 h) and temperature regime (−12 °C) represented threshold conditions for the viability of the leaf apparatus in cultivars susceptible to hypothermic stress. A low capacity for regeneration following such temperature exposure manifested as a further decrease in viability after the stress was removed. All photosynthetic coefficients rapidly decreased as temperatures increased.

Thus, it can be concluded that exposure to −12 °C for 12 h reliably differentiates *O. europaea* L. cultivars into frost-tolerant and susceptible groups.

### 2.4. Enzyme Activity

#### 2.4.1. Moderate Stress (−7 °C)

Complete results are presented in Supplementary File S2. Under control conditions, peroxidase activity ranged from 0.096 to 0.198, polyphenol oxidase (PPO) activity ranged from 0.820 to 2.083 arbitrary units per gram of fresh weight, and catalase activity ranged from 28.9 to 68.0 mL O_2_/g·min. The high activity of the studied enzymes is attributed to cold adaptation processes (+4 °C), which enhance the plants’ frost tolerance. Significant differences in peroxidase and catalase activity between cold-tolerant and susceptible cultivars were not observed, whereas PPO activity was significantly higher in cold-tolerant cultivars.

At the initial stage of exposure to subzero temperatures, an increase in peroxidase and PPO activity was observed, while catalase activity showed an opposite trend. Prolonged exposure to the stress factor led to increased peroxidase activity (Point C), which remained elevated even as the temperature was raised to +4 °C (Point D). In contrast, stabilization of the temperature regime (Point C) resulted in increased catalase activity. The dynamics of PPO activity changes between Points B and D varied across cultivars and showed no clear trend. Notably, during the 24 h recovery period, both peroxidase and PPO activities exhibited a clear tendency to decrease.

#### 2.4.2. Severe Stress (−12 °C)

During the initial stage of low-temperature exposure (Point B), a significant decrease in peroxidase and catalase activity was observed in all cultivars. Cold stress exposure was accompanied by intensified oxidation processes, increased production of ROS, and inhibition of peroxidase and catalase activity.

At the second stage of low-temperature exposure (Point C), when the temperature stabilized, the antioxidant system, including specific redox enzymes such as peroxidase and catalase, was activated in plants to protect against oxidative damage. PPO activity displayed mixed patterns. It decreased in cultivars ‘Coreggiolo’, ‘Razzo’, and ‘Tiflisskaya’, while it significantly increased in cv. ‘Leccino’.

When the stress factor was removed and temperatures stabilized (Point D), an increase in PPO activity and a decrease in catalase activity were observed. The activation of recovery processes (Point E) was characterized by a decline in PPO and catalase activity.

### 2.5. RNA-Seq and Data Processing

The average number of reads after filtering was 17.3 million per library. On average, 63.78% of the reads were uniquely mapped to the *Olea europaea* var. *sylvestris* reference genome, with an average read length of 282 bp. Additionally, the average number of reads corresponding to genomic features was 7.2 million per library.

### 2.6. Differential Gene Expression Under Freezing Stress

The first step involved a general comparative analysis of expression changes. Differentially expressed genes (DEGs) were evaluated by comparing transitions between stages:

B vs. A: Leaf response to rapid cooling.

C vs. B: Leaf response to prolonged exposure to low temperatures (overnight frosts).

D vs. C: Leaf response to warming after brief freezing.

E vs. D: Leaf recovery over 24 h following cold stress.

**Figure 6:**
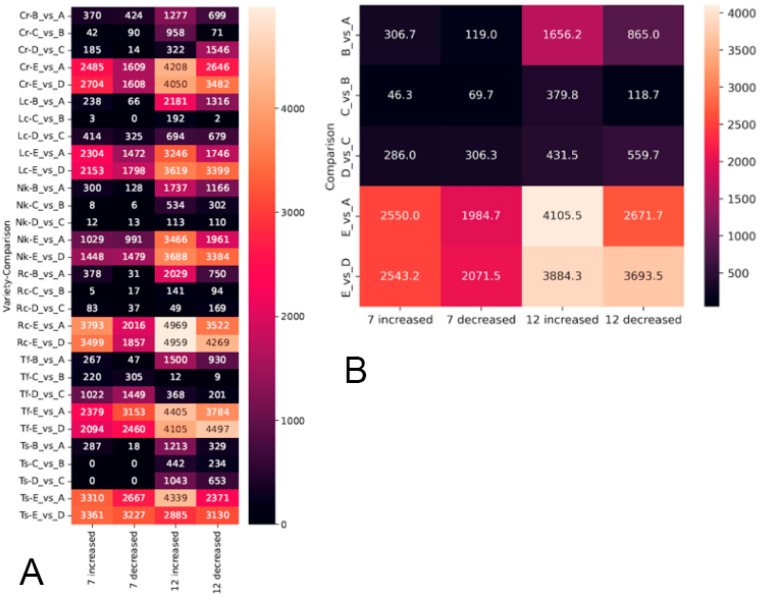
Overall DEGs for all olive cultivars. (**A**) DEG counts by individual cultivars; (**B**) aggregated DEG counts.

We also compared the final recovery stage (E) with the control stage (A) to assess the extent to which leaves returned to their original state. The analysis results are presented in Figure 6.

As shown in Figure 6, several patterns emerge in gene expression changes during cooling. Most often, stage-to-stage DEGs are predominantly represented by genes with increased expression, while genes with decreased expression are less prevalent or equally represented. In Figure 2B, an apparent exception is the warming transition (D vs. C), but the predominance of decreased expression over increased expression is characteristic only for certain cultivars under specific temperature regimes.

The stage with the fewest DEGs is the cold exposure phase (C vs. B), where transcriptional changes are minimal compared with other stages. The most significant changes occur during the 24 h recovery period (E vs. D) and when comparing post-cold-treated leaves with the control (E vs. A).

Tables in Supplementary File S3 list genes whose expression changed across all cultivars within a single comparison (corrected *p*-value < 0.05 and L2FC > 1 for upregulated genes; corrected *p*-value < 0.05 and L2FC < −1 for downregulated genes). These genes were used to construct cluster maps (Supplementary File S4). These genes are limited to the cooling phase (B vs. A), recovery phase (E vs. D), and comparison with the control (E vs. A). The proportion of these genes relative to the total number of DEGs is small, indicating that different mechanisms underlie cold adaptation and recovery from stress across cultivars.

**Figure 7:**
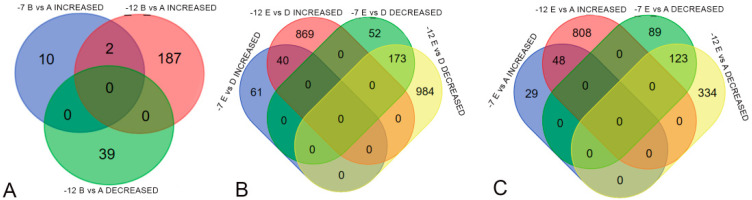
Venn diagrams for overlapping DEGs. (**A**) Overlapping DEGs for the cooling stage (B vs A); (**B**) overlapping DEGs for the recovery stage (E vs D); (**C**) overlapping DEGs for recovery/the control comparison (E vs A).

The genes listed in the specified tables may represent general patterns characteristic of cold shock across all cultivars. A comparison of DEG lists across different temperature conditions revealed that the mechanisms vary due to the cooling temperature (Figure 7).

For the cooling stage (B vs. A), this is particularly evident as there are only two overlapping DEGs with increased expression between B vs. A at −7 °C and B vs. A at −12 °C (Oeu046137.1 and Oeu045100.1). In contrast, in comparisons such as E vs. D and E vs. A, the proportion of shared genes is significantly higher, sometimes exceeding the proportion of distinct DEGs. No gene was found to exhibit decreased expression at one temperature and increased expression at another.

To determine the functional categories of genes that change their expression regardless of cultivar, we performed GO enrichment analysis (GOEA) for the genes listed in the tables from Supplementary File S3. The complete results of this analysis are provided in Supplementary File S5. These genes are grouped into numerous functional categories, many of which have been previously described in the literature as associated with cold response.

### 2.7. Differential Expression in Cold-Susceptible and Cold-Tolerant Cultivars

**Figure 8:**
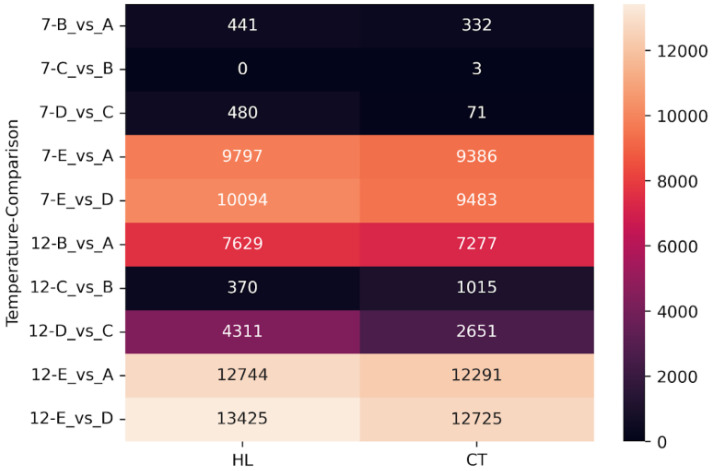
Numbers of DEGs for heat-loving (HL) and cold-tolerant (CT) cultivars.

In the next stage of analysis, we grouped the cultivars based on their cold tolerance into two categories: ‘heat-loving’ (HL) for cold-susceptible cultivars (cv. ‘Leccino’, cv. ‘Razzo’, cv. ‘Coreggiolo’) and ‘cold-tolerant’ (CT) for cold-tolerant cultivars (cv. ‘Nikitskaya-2’, cv. ‘Tossiyskaya’, cv. ‘Tiflisskaya’) and identified expression changes, as shown in Figure 8.

We found that, for the C vs. B comparison, almost no transcriptional changes were detected in the combined groups at −7 °C (zero shared DEGs for the HL group, three shared DEGs for the CT group). For both groups at all temperatures, the most DEG-enriched comparisons were also recovery after freezing (E vs. D) and comparison to the control (E vs. A). The freezing phase (B vs. A), adaptation (C vs. B), and warming (D vs. C) were three categories that showed a significant increase in DEG numbers at −12 °C.

**Figure 9:**
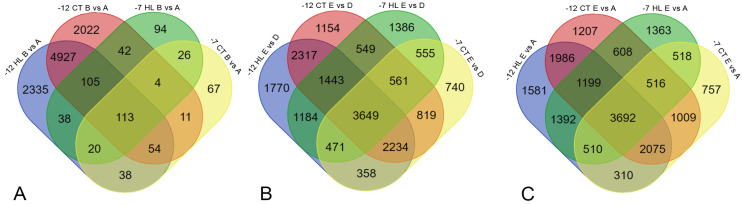
Venn diagrams for overlapping DEGs in cold-tolerant (CT) and heat-loving (HL) cultivars. (**A**) Overlapping DEGs for the cooling stage (B vs A); (**B**) overlapping DEGs for the recovery stage (E vs D), (**C**) overlapping DEGs for recovery/the control comparison (E vs A).

Additionally, we evaluated the overlap of genes across the comparisons B vs. A, E vs. D, and E vs. A (Figure 9).

**Figure 10:**
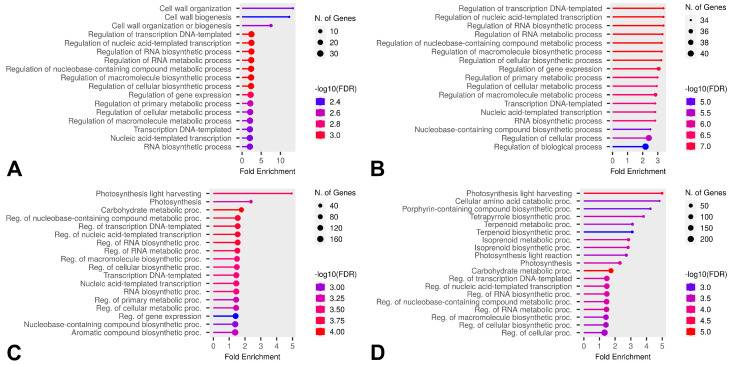
Enriched GO Biological Process categories of unique DEGs in the B vs A comparison across all temperatures and both groups. (**A**) Categories enriched by unique DEGs of cold-tolerant cultivars at −7 °C, (**B**) categories enriched by unique DEGs of heat-loving cultivars at −7 °C, (**C**) categories enriched by unique DEGs of cold-tolerant cultivars at −12 °C, (**D**) categories enriched by unique DEGs of heat-loving cultivars at −12 °C.

**Figure 11:**
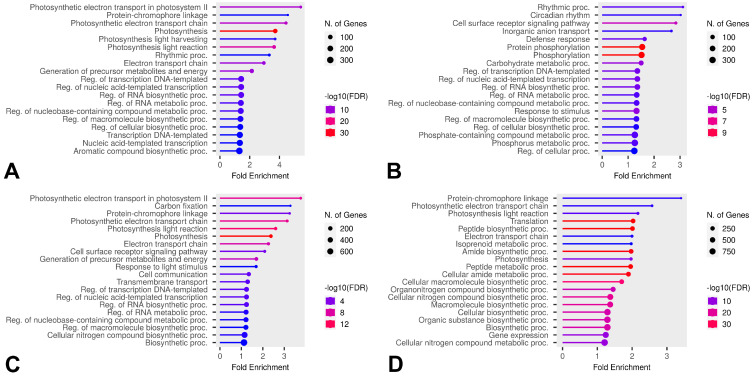
Enriched GO Biological Process categories of unique DEGs in the E vs A comparison across all temperatures and both groups. (**A**) Categories enriched by unique DEGs of cold-tolerant cultivars at −7 °C, (**B**) categories enriched by unique DEGs of heat-loving cultivars at −7 °C, (**C**) categories enriched by unique DEGs of cold-tolerant cultivars at −12 °C, (**D**) categories enriched by unique DEGs of heat-loving cultivars at −12 °C.

**Figure 12:**
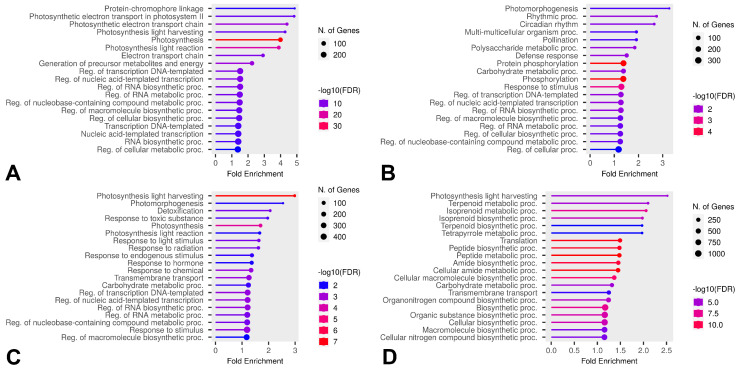
Enriched GO Biological Process categories of unique DEGs in the E vs D comparison across all temperatures and both groups. (**A**) Categories enriched by unique DEGs of cold-tolerant cultivars at −7 °C, (**B**) categories enriched by unique DEGs of heat-loving cultivars at −7 °C, (**C**) categories enriched by unique DEGs of cold-tolerant cultivars at −12 °C, (**D**) categories enriched by unique DEGs of heat-loving cultivars at −12 °C.

**Figure 13:**
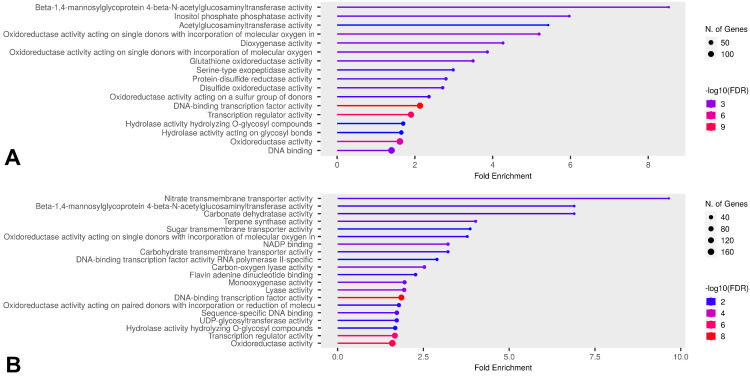
Enriched GO Molecular Function categories of unique DEGs in the B vs A comparison across both groups at −12 °C. (**A**) Categories enriched by unique DEGs of cold-tolerant cultivars, (**B**) categories enriched by unique DEGs of heat-loving cultivars.

Some DEGs overlapped in comparisons, but, based on the Venn diagram results, we further investigated which GO categories were enriched by unique DEGs across all temperatures in the comparisons B vs. A, E vs. A, and E vs. D for both HL and CT groups (Figure 10, Figure 11 and Figure 12). Molecular Function category enrichment analysis was also performed for the B vs. A comparison at −12 °C (Figure 13).

### 2.8. Verification by Quantitative Real-Time PCR

Validation of the results was performed using genes that differ in expression at different stages of freezing in different cultivars. A separate validation was performed for Oeu046137.1 and Oeu045100.1 transcripts in cold-tolerant and heat-loving cultivars separately. The transcripts listed in Supplementary File S6 were selected for the validation. According to Pearson’s correlation coefficient, the results of qPCR correlate with the results of RNA-Seq.

## 3. Discussion

### 3.1. Biochemical and Morphological Processes in Olive Leaves After Freezing

Low subzero temperatures induce oxidative stress due to the overproduction of ROS, including superoxide radicals (O_2_^−^), hydrogen peroxide (H_2_O_2_), and hydroxyl radicals (HO^−^) [31]. Oxidative damage caused by ROS under subzero temperatures has been described in the cultivars ‘Fishomi’ and ‘Roughani’ [32], ‘Leccino’ and ‘Oblica’ [33], ‘Razzo’ and ‘Correggiolo’ [34], and ‘Nikitskaya’ and ‘Ascolano’ [7], among others. The most active antioxidant enzymes protecting olive cells from ROS are catalase (CAT), superoxide dismutase (SOD), peroxidase (PRX), and enzymes in the ascorbate–glutathione cycle [35,36]. Numerous studies have demonstrated the relationship between enzyme activity and the content of reactive oxygen species (ROS) and malondialdehyde (MDA) [3,32,37,38].

Results obtained by other researchers on olive plants have shown that malondialdehyde content is inversely proportional to the activity of peroxidase, catalase, ascorbate peroxidase, and polyphenol oxidase enzymes [39]. This provided a basis for us to assume that, in our study, the observed decrease in catalase and peroxidase activity under severe stress conditions (−12 °C) is due to the overproduction of ROS.

In addition, it has been noted that polyphenol oxidase, apart from its role in scavenging excess ROS, is also consumed in the oxidation of tannins and polyphenols. Furthermore, there are known inhibitors of polyphenol oxidase.

In our studies, the high activity of enzymes at the initial temperature of +4 °C was directly linked to the cold adaptation phase. Similar research was conducted on acclimatized and non-acclimatized plants on the cultivars ‘Bouteillan’, ‘Nostrale di Rigali’, ‘Frantoio’, and ‘Moraiolo’, confirming the significant role of cold adaptation in resistance to low-temperature stress [40].

In our research, cold-tolerant plants demonstrated higher enzyme activity under hypothermic stress compared with non-tolerant genotypes. Specifically, catalase, peroxidase, and polyphenol oxidase activities were directly correlated with the degree of cold tolerance. The antioxidant properties of catalase, peroxidase, and polyphenol oxidase under low-temperature stress in *O. europaea* L. have been extensively documented in the literature [4,30,34,41,42,43].

Photosynthetic metabolism is one of the processes most sensitive to temperature fluctuations. Low temperatures restrict photosynthesis by disrupting all major components of the process, including inhibition of photophosphorylation, electron transport, reduction in the maximum quantum efficiency of Photosystem II (PSII), photochemistry, and carbon fixation. PSII is one of the most susceptible components of the photosynthetic apparatus, and the chlorophyll fluorescence parameter Fv/Fm is a non-invasive measure of PSII activity. It is widely used for monitoring plant responses to environmental changes [44,45,46].

In our studies, a reduction in photosynthetic activity was observed in all cultivars at the initial stage of stress exposure. The strongest response to initial stress was noted in the cultivars ‘Coreggiolo’ and ‘Razzo’. Similar findings were reported in other papers [7,47,48]. At this stage, plants demonstrated disruptions in physiological and biochemical processes and initiated protective responses to stress.

Several authors have also observed reductions in net photosynthetic rate, changes in transpiration and stomatal conductance, PSII damage, and alterations in photosynthetic pigments in olives under low-temperature conditions, highlighting significant differences between olive cultivars [49]. Changes in the Fv/Fm ratio were reported at subzero temperatures, with tolerant genotypes maintaining significantly higher Fv/Fm indices, while non-tolerant cultivars showed reductions, indicating impairment of or damage to PSII reaction centers [7,13,41,49,50,51,52].

In our studies, the reduction in Fv/Fm levels was cultivar specific and significantly more pronounced in cold-susceptible cultivars. A decrease in Fv/Fm at low temperatures under controlled conditions, as well as during winter, has often been observed in evergreen species and attributed to photoprotective mechanisms [53,54,55]. Notably, in studies by D. Guerra et al., olive leaves fully restored Fv/Fm after stress, despite values approaching zero during cold treatment, indicating a strong capacity for photoprotection [13]. In winter, olive trees, like other evergreen species, face a combination of low temperatures and light, which can potentially lead to photoinhibition. Thus, evergreens activate sustained thermal energy dissipation to balance light absorption with reduced light utilization [56].

In our studies, across all experimental conditions simulating hypothermic stress at −7 °C and −12 °C, a decrease in the effective photochemical quantum yield of Photosystem II (Y(II)) was observed. Photoinhibition of photosynthetic productivity occurred in all cultivars regardless of their frost tolerance. However, in the cold-tolerant cultivars ‘Nikitskaya-2’, ‘Tiflisskaya’, and ‘Tossiyskaya’, Y(II) values returned to normal after the stress factor was removed. In contrast, cultivars ‘Coreggiolo’, ‘Leccino’, and ‘Razzo’ exhibited damage to chlorophyll–protein complexes. Similar results were reported by D. Guerra et al. and T. Gubanova et al. [7,13].

In the work of Sobieszczuk-Nowicka et al., the viability index Rfd (Relative Fluorescence Decrease) was identified as the earliest parameter to correlate well with the cessation of photosynthesis, reflecting the interaction between light-dependent reactions utilizing photosynthetically active radiation and dark phase reactions [57]. In our studies, at temperatures reduced to −12 °C, this parameter decreased, indicating a disruption in the balance between photochemical reactions in the thylakoids and the rate of enzymatic reactions in the chloroplast stroma. According to Lichtenthaler et al., under changes in photosynthetically active radiation, the viability coefficient can be considered a measure of the potential activity of photosynthesis and an indicator of stress levels [58,59]. A positive linear correlation between Rfd values and the intensity of photosynthetic CO_2_ assimilation was identified. Rinderle et al. noted that Rfd values around 2.5 or higher indicate high photosynthetic activity, while values below 1.0 suggest that CO_2_ assimilation is severely suppressed [60].

In our research, the increase in Rfd in cold-tolerant cultivars 24 h after the cessation of the stress factor (at −12 °C) indicates the initiation of repair processes, whereas in non-tolerant cultivars the continued decrease in the viability index suggests that vital thresholds have been reached. Significant damage to the light-harvesting complexes in non-tolerant cultivars is also indicated by the increase in non-regulated quantum energy losses (Y(NPQ)). These findings are consistent with studies by Lichtenthaler et al., which demonstrated a reduction in Rfd under external stress conditions [58,59,61].

### 3.2. Differential Expression During Freezing and Recovery of Leaves

The observed differences in the number of DEGs highlight how significantly transcriptional load varies across different stages of freezing and recovery. These findings led us to focus on two key processes described above: leaf cooling to reach the target temperature (freezing process, B vs. A) and leaf recovery after cold shock (warming process, E vs. D).

The predominance of DEGs in the comparisons B vs. A and E vs. D indicates that cooling and recovery are the most complex and transcriptionally demanding processes, requiring the involvement of a large number of genes and numerous molecular–genetic pathways. Furthermore, at −12 °C, molecular mechanisms in leaves were activated more intensely at every stage of cooling examined.

For the identification of overall DEGs without differentiating between cold-tolerant and heat-loving cultivars, the GO category enrichment patterns largely aligned with cold response observations in other plants. At the onset of cooling under non-critical freezing conditions, genes associated with biosynthetic and metabolic processes showed altered expression. At the same temperature, during recovery from cold stress, genes involved in defense responses and stimulus response were upregulated, while genes related to photomorphogenesis exhibited decreased expression. This is expected, as photosynthesis slows under cold conditions, halting the development of associated structures. At extreme freezing temperatures, during recovery, categories associated with cold response are enriched, including calcium signaling [62], ethylene molecular pathways [63], photosynthesis [64], and circadian rhythms [65].

Differences in enriched processes and categories were also notable when comparing the final and initial stages at different temperatures. At −7 °C, recovery-stage leaves showed upregulated expression of genes associated with carbohydrate metabolism, stimulus responses, and certain biosynthetic processes, while genes related to the regulation of various biosynthetic processes and circadian rhythms exhibited downregulated expression. At −12 °C, upregulated genes were linked to isoprenoid and lipid biosynthesis, calcium signaling, negative growth regulation, and ethylene molecular pathways. Downregulated genes were associated with photomorphogenesis, stimulus responses, and other biosynthetic processes.

As is evident from these categories, exposure to extreme temperatures activates or prolongs the action of a greater number of known cold-response mechanisms.

Two transcripts, Oeu046137.1 (GATA transcription factor 5-like) and Oeu045100.1, were expressed in all cultivars and at all temperatures during cooling according to our data. Primary cold-response genes, such as *CBF*, are active during the first hours of cooling (with a functional window of 1–3 h [66]), and thus their differential expression is not visible at the lowest cooling point. GATA transcription factors have been extensively studied in the context of abiotic stress responses in various plants, but they are mostly associated with salt and drought stress responses [67,68,69], with a few cases noting their role in cold responses [70]. Previously, reduced expression of GATA transcription factor 21-like was observed in olives during infection by *Spilocaea oleagina* [71]. No functional data are available for Oeu045100 or its homologs, but it is plausible that this gene is also a transcription factor linked to freezing stress response. The further investigation of these two transcripts is needed to confirm the association.

### 3.3. DEGs in Cold-Tolerant and Cold-Susceptible Cultivars

The quantitative comparison of DEGs between cold-tolerant and cold-susceptible (‘heat-loving’) cultivars revealed several patterns. For the most part, the level of differential expression was similar across stages in both groups. However, a key difference persisted under various temperature conditions. During the slow recovery of temperature to baseline (D vs. C), heat-loving cultivars exhibited higher differential expression than cold-tolerant ones. Conversely, during adaptation to extreme cold temperatures (C vs. B) before recovery, heat-loving cultivars showed lower levels of differential expression.

We hypothesize that this abrupt difference in expression levels during warming could be due to slowed transcriptional processes during the prior acclimation stage. The combination of low temperatures and a limited presence of cryoprotective agents may cause slower internal responses in the leaves of heat-loving cultivars. Consequently, regulatory gene expression surges during warming as the plants attempt to minimize cold-induced damage. In cold-tolerant cultivars, damage and reaction slowdowns during the cold acclimation stage are less pronounced due to mechanisms underlying their cold tolerance.

During recovery (E vs. D) and comparisons with the control (E vs. A), the distribution of shared and unique DEGs across stages was almost identical. Nevertheless, in all cases, the number of shared DEGs between HL and CT groups was higher than the number of unique DEGs at −12 °C. This suggests that predominantly similar mechanisms are activated during extreme freezing, while intergroup differences are more noticeable quantitatively during moderate freezing at −7 °C.

The enrichment analysis of categories for unique genes revealed the processes associated with genes that differ at various temperatures in cold-tolerant and heat-loving cultivars. During the cooling phase, both cold-tolerant and cold-susceptible cultivars exhibit differential expression in regulative GO categories; however, the expression of genes related to cell wall formation differs at −7 °C for cold-tolerant cultivars. The alteration in the expression of genes related to oxidative stress in cultivars at extreme temperatures (−12 °C) suggests that these genes might contribute to the optimal responses in leaves by reducing excessive ROS activity.

During the recovery phase after cooling, both cold-tolerant and cold-susceptible cultivars showed differential expression associated with photosynthesis. As mentioned earlier, cold stress negatively impacts photosynthesis, and we hypothesize that the unique photosynthesis-related DEGs might help restore the photosynthetic apparatus more quickly or adapt it to new conditions. At −7 °C, cold-susceptible cultivars also exhibit a high level of differential expression in the phosphorylation category. Protein phosphorylation is associated with protein activation and inhibition. Cold-susceptible cultivars display specific levels of expression of regulatory genes during recovery, with enrichment in the phosphorylation category observed both during recovery and when comparing the final and initial stages.

### 3.4. Epigenetic Modifications in Native Cold-Tolerant Cultivars

Plant cold responses often involve DNA methylation and other epigenetic changes that reprogram gene expression without altering DNA sequences. The cold-tolerant cultivars in this study (cv. ‘Nikitskaya-2’, cv. ‘Tossiyskaya’, and cv. ‘Tiflisskaya’) originate from much colder regions than the cold-susceptible ones (cv. ‘Leccino’, cv. ‘Razzo’, and cv. ‘Coreggiolo’), which may affect the reaction to cold stress on the epigenetic and, consequently, transcriptomic level and be the possible explanation for enhanced cold tolerance. Olive cuttings from the same cultivar grown in distinct climates might carry forward an epigenetic imprint of their environment, although concrete studies in *O. europaea* are not yet available [4].

However, there are examples of epigenetic changes under cold exposure in other tree species. For example, a notable case of an environment-driven methylation change is seen in the rubber tree (*Hevea brasiliensis*) [72]. *Hevea* clones grown in colder high-altitude regions developed greater cold tolerance despite very low genetic variation among cultivars. In this case, it was found that cold exposure caused active DNA demethylation in these trees, especially in the promoters of crucial cold-regulation genes (*HbICE1*, *HbCBFs*). Cold-tolerant *Hevea* clones showed a significant loss of DNA methylation at these loci, correlating with their increased expression and stress tolerance.

In long-lived woody plants like *O. europaea*, for breeding and selection purposes there is interest in whether epigenetic adaptations to climate can be passed to offspring or maintained long term. In Norway spruce (*Picea abies*), the temperature during seed development programs an epigenetic memory in the embryos that influences the seedlings’ growth cycle for years after germination [73]. Spruce seeds matured in a warm vs. cool environment produce genetically identical seedlings (same genotype) that nevertheless show different timings of bud set and bud burst, an adaptation to the parental temperature regime. This was traced to differential DNA methylation imprinted during embryogenesis. It shows a transgenerational effect, since the offspring carry a memory of parental environmental conditions.

## 4. Materials and Methods

### 4.1. Plant Material

Leafy shoots from mature olive (*Olea europaea* L.) trees growing in the collection of the Nikitsky Botanical Garden—National Scientific Center (NBG-NSC, Yalta, Crimea) were used in this study. The plants were cultivated under identical climatic, soil, and agronomic conditions. The sampling and experiment locations were identified using GPS coordinates (44°51′24′ N, 34°23′94′ E, 200 m above sea level). This region has a subtropical climate characterized by dry, hot summers and wet winters, with precipitation concentrated mainly in autumn and winter. The average annual sunshine duration is 2285 h, with 188 mm of rainfall from May to September and a total annual precipitation of 595 mm. The absolute minimum temperature in the area is −14.6 °C, with an average annual temperature of +12.4 °C and average annual humidity of 67%. The soil is a brown and slightly calcareous heavy loam on clay shales and limestones. The trees were planted at a spacing of 5 × 5 m, with a planting density of 400 trees per hectare, on a south-facing slope.

Six olive cultivars were selected for the study. Three (cv. ‘Leccino’, cv. ‘Razzo’, and cv. ‘Coreggiolo’) are considered cold susceptible under the conditions of the southern coast of Crimea, and three (cv. ‘Nikitskaya-2’, cv. ‘Tossiyskaya’, and cv. ‘Tiflisskaya’) are considered cold tolerant. Three biological replicates were sampled for each cultivar.

### 4.2. Leaf Freezing Procedure

**Figure 14:**
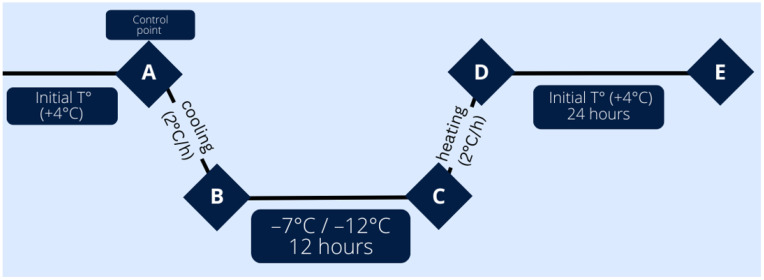
The scheme for the leaf freezing experiment.

The general experimental scheme is shown in Figure 14. Low-temperature stress conditions (−7 °C and −12 °C) were simulated using a TTC 256 Memmert climate test chamber (Memmert, Germany). The temperature reduction began at +4 °C, with a cooling and warming gradient of 2 °C/hour, as described in [74,75]. Leaves were sampled at five different stages:

Stage A (Control): Leaves sampled at the initial temperature before cold exposure.

Stage B: Leaves sampled immediately after reaching the target temperature (−7 °C/−12 °C).

Stage C: Leaves sampled after 12 h of exposure to the target temperature (−7 °C/−12 °C).

Stage D: Leaves sampled immediately after returning to the initial temperature.

Stage E: Leaves sampled after 24 h of recovery at the initial temperature.

This experimental design allowed for the assessment of both the immediate and recovery responses of olive leaves to moderate and extreme freezing stress.

### 4.3. Photosynthetic Apparatus Analysis

Chlorophyll fluorescence measurements were conducted using a MINI-PAM II portable pulse fluorimeter (Heinz Walz, Effeltrich, Germany). Leaves were dark adapted for 30 min before fluorescence parameter measurements.

The following calculated coefficients were used in the study:

1. Maximum photochemical quantum yield of PSII: Fv/Fm;
2. Photosynthetic activity: PA = (Fm − Fs)/Fm;
3. Fluorescence decrease ratio: Rfd = (Fs − Fm)/Fs;
4. Effective photochemical quantum yield of PSII: Y(II) = (Fm’ − Fs)/Fm’;
5. Quantum yield of regulated non-photochemical energy dissipation in PSII: Y(NPQ) = Fs/Fm’ − Fs/Fm;
6. Quantum yield of non-regulated non-photochemical energy dissipation in PSII: Y(NO) = Fs/Fm.

### 4.4. Structural Analysis

To reveal leaf tissue damage after freezing, the hand-sectioning method was applied. Transverse sections prepared by hand were placed in a Petri dish or on a glass slide and examined using an SMZ 745T stereomicroscope (Nikon, Nanjing, China) equipped with a DC29111251 digital camera (View Solutions, Rancho Cucamonga, CA, USA) and ImageView v. software. x64, 3.7.10121.20171030.

To determine the influence of freezing temperatures on the leaf tissue and cell reaction, organ middle portions were cut out and immediately fixed in 2.5% glutaraldehyde on 0.1 M cacodylate buffer (pH 7.2) at room temperature for 3 h, washed twice with the same buffer, postfixed in 1% OsO4 for 3 h, dehydrated in a graded alcohol series and acetone, and embedded in an Epon–Araldite epoxy resin mixture. Semithin (1–2 µm) transverse sections were obtained using an Ultracut E ultratome (Reichert, Vienna, Austria). Staining was carried out with an aqueous solution of toluidine blue (0.12%). Slides were investigated on CX41 (Olympus, Japan) microscopes equipped with an SC 50 camera (Olympus, Munich, Germany) and CellSens Imaging Software v. 1.17.

To analyze the ultrastructural organization of the mesophyll cells, leaf portions embedded in epoxy resin were also used for an anatomical assay. Transverse ultrathin sections (50–60 nm) were obtained on a Leica EM UC6 (Leica, Munich, Germany) ultramicrotome, contrasted with UranyLess EM Stain (Electron Microscopy Sciences, Hatfield, PA, USA) and lead citrate, and examined on a Tecnai G2 Spirit Bio TWIN (FEI, Eindhoven, Netherlands) [76]. Cells with detached membranes were revealed in a digital image using UTHSCSA Image-Tool software v. 3.00. Samples were checked for a normal distribution, and either the t-test or the U-criterion test was used.

### 4.5. Enzyme Activity

#### 4.5.1. Catalase Activity

Catalase activity was measured using a titrimetric method. A plant tissue sample (0.2 g) was ground in a mortar with water, transferred to a 50 mL volumetric flask, filled to the mark, and filtered. Five milliliters of the filtrate was incubated with 5 mL of a 0.3% hydrogen peroxide solution for 10 min. The reaction was stopped by adding 5 mL of 10% sulfuric acid. The remaining hydrogen peroxide was titrated with a 0.05 N potassium permanganate solution. A control sample was prepared similarly but preheated in a boiling water bath for 5 min. Catalase activity is expressed as the amount of O_2_ produced by the enzyme per minute per gram of fresh weight.

#### 4.5.2. Peroxidase Activity

Peroxidase activity was measured colorimetrically based on the rate of benzidine oxidation [77]. A plant material sample (0.5 g) was ground in a mortar with an acetate buffer solution (pH = 4.7), transferred to a 25 mL volumetric flask, filled with water to the mark, and filtered. In a 2 cm quartz cuvette, 2 mL of the filtrate, 2 mL of benzidine solution, and 2 mL of water were mixed. For the experimental sample, 2 mL of a 3% hydrogen peroxide solution was added instead of water. Measurements were taken with a KFK-2 photoelectric colorimeter, recording the time for the optical density to change from 0 to 0.1 at a wavelength of 590 nm. Peroxidase activity is expressed in optical density units per gram of fresh weight per second.

#### 4.5.3. Polyphenol Oxidase (PPO) Activity

PPO activity was measured colorimetrically in the presence of pyrocatechin and p-phenylenediamine [77]. A plant material sample (0.5 g) was ground in a mortar with a phosphate buffer solution (pH = 7.4), transferred to a 25 mL volumetric flask, filled with water to the mark, and filtered. In a 2 cm quartz cuvette, 2 mL of the filtrate, 2 mL of water, and 2 mL of p-phenylenediamine were mixed. For the experimental sample, 2 mL of a pyrocatechin solution was added instead of water. Measurements were taken with a KFK-2 photoelectric colorimeter, recording the optical density at a wavelength of 590 nm every 30 s for 3 min. PPO activity is expressed in optical density units per gram of fresh weight per second.

### 4.6. RNA Extraction, Library Preparation, and Sequencing

A total of 162 samples were analyzed ((6 cultivars × 3 biological replicates × 4 stages) × 2 temperature regimes, plus 6 × 3 (control samples)). After cold exposure, all collected samples were immediately placed in an IntactRNA stabilizer (Evrogen, Moscow, Russia) and stored at −80 °C. RNA extraction was performed in triplicate using the RNeasy Plant Mini Kit (Qiagen, Hilden, Germany). Plant material samples (100–150 mg) were pulverized using a mortar and pestle in the presence of liquid nitrogen, followed by total RNA extraction according to the RNeasy Plant Mini Kit protocol. RNA yield was quantified using a Qubit fluorometer (Invitrogen, Carlsbad, CA, USA) and the Qubit™ RNA HS Assay Kit.

Barcoded RNA-Seq libraries were prepared using the MGIEasy Fast RNA Library Prep Set (MGI, Shenzhen, China) according to the manufacturer’s protocol. mRNA isolation from total RNA was performed using the NEBNext^®^ Poly(A) mRNA Magnetic Isolation Module (New England BioLabs, Ipswich, MA, USA). Libraries were sequenced on the high-throughput DNBSEQ-G400 sequencer (MGI, Shenzhen, China) using the High-throughput Sequencing Set (PE150, 540 Gb) reagents with paired-end reads (2 × 150 bp) at the National Research Center “Kurchatov Institute” (Moscow, Russia).

### 4.7. Bioinformatic Analysis

RNA reads were filtered for quality (phred > 20), and library adapters were trimmed using Cutadapt software (version 4.4). Reads were then mapped to the *Olea europaea* var. *sylvestris* genome (GCA_002742605.1) using STAR (version 2.7.11). Gene models representing non-overlapping exonic fragments were obtained from the RefSeq database. For each gene, the total coverage by mapped reads in each sample was determined using bedtools coverage (version 2.30.0), and gene expression was quantified with HTSEQ (version 2.0.5).

Differential gene expression analysis was performed using the R package DESeq2 [78]. Genes were filtered based on an adjusted *p*-value threshold of < 0.05 to identify statistically significant differential expression. Gene Ontology (GO) terms were assigned to the genes using biomaRt [79], and GO pathway enrichment analysis was conducted with the topGO package [80]. Final data analysis and result visualization were carried out in Python ver. 3.11.10 using the pandas [81] and seaborn [82] libraries. For further analysis and visual representation, the module L2FC > 1, an adjusted *p*-value < 0.05, a threshold of a minimum of 5 annotated genes in a category, and average expression (baseMean) > 10 were used.

### 4.8. Verification by Quantitative Real-Time PCR

Ten DEGs were selected for quantification. Each reaction was carried out using the CFX96 (Bio-Rad, Hercules, CA, USA) optical reaction module, One-Tube RT-PCR SYBR Mix (Evrogen, Moscow, Russia), and specific primers. For normalization of the data, the olive *GAPDH* gene was used as an endogenous reference [83]. Technical replicates were performed for each gene. Based on the qPCR data, gene expression was calculated from Cq values for each gene and normalized to the Cq values of the *GAPDH* reference gene using the pcr R package and pcr_analyze() [84]. The gene expression correlation between RT-qPCR and RNA-seq data was estimated using Pearson’s correlation coefficient.

## 5. Conclusions

We conducted a multi-component comparative analysis of two groups of *Olea europaea* L. cultivars: cold tolerant (cv. ‘Nikitskaya-2’, cv. ‘Tossiyskaya’, and cv. ‘Tiflisskaya’) and cold susceptible (cv. ‘Leccino’, cv. ‘Razzo’, and cv. ‘Coreggiolo’). The physiological and biochemical differences between the two groups were confirmed through experiments.

In this study, it was shown that cold-tolerant and cold-susceptible cultivars had more unique DEGs at −7 °C, while at −12 °C they shared more common DEGs. The detected differences in unique DEGs between the two groups clustered into distinct functional categories similar to the ones found in previous studies on cold tolerance in plants. Two overlapping DEGs with increased expression were found in all cultivars during initial freezing.

## Figures and Tables

**Table 1:** Climatic conditions on the southern coast of Crimea (1991–2020).

Month	Temperature, °C				Air Humidity, %	Total Precipitation, mm
	Average	Min	Max			
January	3.7	−12.4	17.0		77	78
February	3.6	−11.9	25.2		75	61
March	6.0	−5.3	23.2		72	54
April	10.7	−5.5	27.5		67	31
May	16.0	4.0	31.6		67	33
June	20.9	8.8	35.6		63	41
July	24.2	11.6	37.8		58	34
August	24.4	13.0	39.0		56	37
September	19.3	8.0	33.4		62	41
October	14.0	−0.3	32.2		70	51
November	9.0	−6.3	24.7		74	60
December	5.5	−8.4	20.4		77	87
Average temperature for the year	Absolute min temperature	Average absolute min temperature	Absolute max temperature	Average absolute max temperature	Average humidity for the year	Total precipitation for the year
13.1	−12.4	−7.1	39.0	34.8	68	609

**Table 2:** Percentage of the cell with a detached membrane after exposure to freezing temperatures. *, statistically significant differences between two parameters in a line; t-criterion; n = 9; *p* < 0.05.

Temperature	Cells	‘Corregiolo’	‘Nikitskaya-2’
−7 °C	Palisade	0.98 ± 0.04	0.30 ± 0.01
	Spongy mesophyll	4.83 ± 0.20	4.64 ± 0.28
−12 °C	Palisade	5.88 ± 0.55	4.30 ± 0.21
	Spongy mesophyll	32.49 ± 1.67	19.44 ± 0.86 *

## Data Availability

The raw sequence data have been deposited into the National Center for Biotechnology Information (NCBI) repository and the accession number is PRJNA1190217.

## Supplementary Materials

The following supporting information can be downloaded at: https://www.mdpi.com/article/10.3390/ijms26093934/s1, File S1: Photosynthetic activity measurements; File S2: Enzyme activity measurements; File S3: DEGs in all cultivars; File S4: Clustermaps for DEGs in all cultivars; File S5: Gene Ontology enrichment data; File S6: Transcripts for qPCR.
