# Supplementary material for: Freeze-Dependent Physiological and Transcriptional Changes in Olea europaea L. Cultivars with Different Cold Resistances
Source: Int J Mol Sci. 2025 Apr 22;26(9):3934. doi: 10.3390/ijms26093934 (PMC12071819; doi:10.3390/ijms26093934)
Supplement: Supplementary file 1 [file ijms-26-03934-s001.zip › Supplementary/Supplementary File S1.docx]

**Parameters of photosynthetic activity of European olive, determined by the criteria of chlorophyll fluorescence induction after exposure to low-temperature stress (temperature –7°C)**

| Cultivar | T °C | Photosynthetic activity coefficients | | | | | |
| --- | --- | --- | --- | --- | --- | --- | --- |
|  |  | *Fv/Fm* | *PA* | *Rfd* | *Y(NPQ)* | *Y(NO)* | *Y(II)* |
| Coreggiolo | control | 0.702 | 0.668 | 2,008 | 0.273 | 0.333 | 0.394 |
|  | - 7 | 0.643 | 0.664 | 1,976 | 0.313 | 0.336 | 0.351 |
|  | -7(12) | 0.721 | 0.698 | 2,309 | 0.367 | 0.303 | 0.330 |
|  | +4 | 0.718 | 0.687 | 2,204 | 0.389 | 0.335 | 0.276 |
|  | +4(24) | 0.704 | 0.668 | 2,113 | 0.393 | 0.301 | 0.306 |
| Leccino | control | 0.517 | 0.583 | 1,401 | 0.282 | 0.433 | 0.284 |
|  | - 7 | 0.311 | 0,450 | 0.817 | 0.557 | 0.244 | 0.198 |
|  | -7(12) | 0, 521 | 0, 585 | 1,411​​ | 0, 542 | 0, 287 | 0.1 71 |
|  | +4 | 0,520 | 0.583 | 1,390 | 0.537 | 0.319 | 0.144 |
|  | +4(24) | 0.604 | 0.613 | 1,467 | 0.390 | 0.357 | 0.253 |
| Razzo | control | 0.667 | 0.696 | 2,291 | 0.345 | 0.312 | 0.343 |
|  | - 7 | 0.488 | 0, 4 37 | 1,756 | 0.359 | 0.393 | 0.248 |
|  | -7(12) | 0, 650 | 0.6 90 | 1, 9 5 3 | 0, 549 | 0, 303 | 0, 1 4 7 |
|  | +4 | 0.646 | 0.682 | 1,781 | 0,470 | 0.394 | 0.136 |
|  | +4(24) | 0.639 | 0,680 | 1,667 | 0.441 | 0.354 | 0.205 |
| Nikitskaya-2 | control | 0 ,668 | 0.638 | 1,760 | 0.283 | 0.383 | 0.334 |
|  | - 7 | 0.562 | 0.637 | 1,753 | 0.313 | 0.389 | 0.298 |
|  | -7(12) | 0, 689 | 0.6 52 | 1.7 83 | 0.3 87 | 0.3 70 | 0.2 43 |
|  | +4 | 0,690 | 0.654 | 1,780 | 0,470 | 0.330 | 0,200 |
|  | +4(24) | 0.734 | 0,660 | 2,012 | 0.330 | 0.336 | 0.334 |
| Tiflisskaya | control | 0.539 | 0.648 | 1,838 | 0.245 | 0.361 | 0.394 |
|  | - 7 | 0.415 | 0.562 | 1,281 | 0.272 | 0.439 | 0.289 |
|  | -7(12) | 0,680 | 0.651 | 1,865 | 0.370 | 0.353 | 0.277 |
|  | +4 | 0.674 | 0,650 | 1,870 | 0.462 | 0.371 | 0.167 |
|  | +4(24) | 0.736 | 0.679 | 2,007 | 0.454 | 0.233 | 0.313 |
| Tossiyskaya | control | 0.649 | 0.646 | 1,822 | 0.296 | 0.360 | 0.344 |
|  | - 7 | 0.455 | 0.615 | 1,600 | 0.315 | 0.422 | 0.263 |
|  | -7(12) | 0, 65 5 | 0.6 47 | 1, 838 | 0, 49 5 | 0, 376 | 0, 129 |
|  | +4 | 0.671 | 0.649 | 1,844 | 0.502 | 0.374 | 0.124 |
|  | +4(24) | 0.722 | 0.653 | 2,064 | 0.411 | 0.268 | 0.321 |

**Parameters of photosynthetic activity of European olive, determined by the criteria of chlorophyll fluorescence induction after exposure to low-temperature stress (temperature –12°C)**

| Cultivar | T °C | Photosynthetic activity coefficients | | | | | |
| --- | --- | --- | --- | --- | --- | --- | --- |
|  |  | *Fv/Fm* | *PA* | *Rfd* | *Y(NPQ)* | *Y(NO)* | *Y(II)* |
| Coreggiolo | control | 0.702 | 0.668 | 2,008 | 0.273 | 0.333 | 0.394 |
|  | -12 | 0.377 | 0.493 | 0.972 | 0.280 | 0.605 | 0.116 |
|  | -12(12) | 0.406 | 0.341 | 0.517 | 0.226 | 0.678 | 0.096 |
|  | +4 | 0.404 | 0.340 | 0.512 | 0.174 | 0.753 | 0.073 |
|  | +4(24) | 0.337 | 0.312 | 0.406 | 0.101 | 0.865 | 0.034 |
| Leccino | control | 0.517 | 0.583 | 1,401 | 0.282 | 0.433 | 0.284 |
|  | -12 | 0.499 | 0.537 | 1,002 | 0.376 | 0.487 | 0.137 |
|  | -12(12) | 0.424 | 0.343 | 0.522 | 0.244 | 0,650 | 0.106 |
|  | +4 | 0.452 | 0.351 | 0.509 | 0.188 | 0.715 | 0.097 |
|  | +4(24) | 0.344 | 0.338 | 0.413 | 0.122 | 0.849 | 0.029 |
| Razzo | control | 0.667 | 0.696 | 2,291 | 0.345 | 0.312 | 0.343 |
|  | -12 | 0.313 | 0.356 | 0.577 | 0.206 | 0.679 | 0.115 |
|  | -12(12) | 0.211 | 0.206 | 0.381 | 0.106 | 0.838 | 0.056 |
|  | +4 | 0.188 | 0.194 | 0.375 | 0.099 | 0.851 | 0,050 |
|  | +4(24) | 0.078 | 0.088 | 0.212 | 0.062 | 0.914 | 0.024 |
| Nikitskaya-2 | control | 0 ,668 | 0.638 | 1,760 | 0.283 | 0.383 | 0.334 |
|  | -12 | 0,640 | 0.637 | 1,749 | 0.386 | 0.355 | 0.259 |
|  | -12(12) | 0.6 22 | 0.6 38 | 1, 658 | 0, 403 | 0.3 79 | 0.2 18 |
|  | +4 | 0.656 | 0.638 | 1,750 | 0.449 | 0.334 | 0.217 |
|  | +4(24) | 0.701 | 0.669 | 1,974 | 0.471 | 0.327 | 0.202 |
| Tiflisskaya | control | 0.539 | 0.648 | 1,838 | 0.245 | 0.361 | 0.394 |
|  | -12 | 0.344 | 0.527 | 1,113 | 0.266 | 0.473 | 0.261 |
|  | -12(12) | 0.373 | 0.545 | 1,198 | 0.279 | 0.455 | 0.267 |
|  | +4 | 0.502 | 0,600 | 1,322 | 0.397 | 0.405 | 0.198 |
|  | +4(24) | 0.664 | 0.612 | 2,013 | 0.469 | 0.343 | 0.188 |
| Tossiyskaya | control | 0.649 | 0.646 | 1,822 | 0.296 | 0.360 | 0.344 |
|  | -12 | 0.627 | 0.606 | 1,703 | 0.433 | 0.358 | 0.209 |
|  | -12(12) | 0, 599 | 0,60 0 | 1, 687 | 0, 502 | 0.3 77 | 0, 121 |
|  | +4 | 0.601 | 0,600 | 1,689 | 0.517 | 0.367 | 0.116 |
|  | +4(24) | 0.651 | 0.648 | 1,997 | 0.506 | 0.348 | 0.146 |
