# Supplementary material for: Freeze-Dependent Physiological and Transcriptional Changes in Olea europaea L. Cultivars with Different Cold Resistances
Source: Int J Mol Sci. 2025 Apr 22;26(9):3934. doi: 10.3390/ijms26093934 (PMC12071819; doi:10.3390/ijms26093934)
Supplement: Supplementary file 1 [file ijms-26-03934-s001.zip › Supplementary/Supplementary File S2.docx]

I experiment (-12)

Peroxidase activity, conventional units/g s

| Cultivars | **A (Control)** | **B** | **C** | **D** | **E** |
| --- | --- | --- | --- | --- | --- |
| 1 (Coreggiolo) | 0.096 | 0,071 | 0.105 | 0.161 | 0.136 |
| 2 (Leccino) | 0.145 | 0,120 | 0.234 | 0.091 | 0.125 |
| 4 (Razzo) | 0.198 | 0.161 | 0.172 | 0.184 | 0.105 |
| 3 (Nikitskaya-2) | 0.175 | 0.132 | 0.125 | 0.147 | 0.167 |
| 5 (Tiflisskaya) | 0.189 | 0.093 | 0.156 | 0.152 | 0,130 |
| 6 (Tossiyskaya) | 0.179 | 0,080 | 0.122 | 0.156 | 0.089 |

Polyphenoloxidase activity, conventional units/g s

| Cultivars | **A (Control)** | **B** | **C** | **D** | **E** |
| --- | --- | --- | --- | --- | --- |
| 1 (Coreggiolo) | 0.820 | 0.952 | 0,800 | 1,250 | 0.634 |
| 2 (Leccino) | 0.392 | 0.588 | 1,613 | 0.802 | 1,271 |
| 4 (Razzo) | 1,087 | 1,316 | 1,000 | 1,562 | 0.535 |
| 3 (Nikitskaya-2) | 1,250 | 0.962 | 0.952 | 1,299 | 1,136 |
| 5 (Tiflisskaya) | 1,818 | 2,000 | 0.794 | 1,471 | 0.645 |
| 6 (Tossiyskaya) | 2,083 | 1,667 | 1,663 | 2,174 | 1,923 |

Catalase activity, ml O _2_ /g min

| Cultivars | **A (Control)** | **B** | **C** | **D** | **E** |
| --- | --- | --- | --- | --- | --- |
| 1 (Coreggiolo) | 66.7 | 14.4 | 39.5 | 15.8 | 39.9 |
| 2 (Leccino) | 68.0 | 35.3 | 75.6 | 45.3 | 13.6 |
| 4 (Razzo) | 38.7 | 38.2 | 56.9 | 38.5 | 24.6 |
| 3 (Nikitskaya-2) | 33.8 | 36.5 | 48.9 | 52.4 | 56.5 |
| 5 (Tiflisskaya) | 62.9 | 56.1 | 70.5 | 62.6 | 35.7 |
| 6 (Tossiyskaya) | 28.9 | 31.4 | 32.3 | 20.6 | 15.7 |

II experiment (-7)

Peroxidase activity, conventional units/g s

| Cultivars | **A (Control)** | **B** | **C** | **D** | **E** |
| --- | --- | --- | --- | --- | --- |
| 1 (Coreggiolo) | 0.096 | 0,110 | 0,250 | 0.214 | 0.167 |
| 2 (Leccino) | 0.145 | 0.123 | 0.316 | 0.352 | 0.191 |
| 4 (Razzo) | 0.198 | 0.307 | 0.311 | 0.309 | 0.284 |
| 3 (Nikitskaya-2) | 0.175 | 0.229 | 0.340 | 0.333 | 0.205 |
| 5 (Tiflisskaya) | 0.189 | 0.202 | 0.229 | 0.253 | 0.240 |
| 6 (Tossiyskaya) | 0.179 | 0.272 | 0.379 | 0.312 | 0.360 |

Polyphenoloxidase activity, conventional units/g s

| Cultivars | **A (Control)** | **B** | **C** | **D** | **E** |
| --- | --- | --- | --- | --- | --- |
| 1 (Coreggiolo) | 0.820 | 1,020 | 1,667 | 1,205 | 0.769 |
| 2 (Leccino) | 0.392 | 1,316 | 2,000 | 0.758 | 0.725 |
| 4 (Razzo) | 1,087 | 0.654 | 0.613 | 1,000 | 0.935 |
| 3 (Nikitskaya-2) | 1,250 | 1,515 | 1,153 | 1,741 | 1,639 |
| 5 (Tiflisskaya) | 1,818 | 2,000 | 2,128 | 2,898 | 2,431 |
| 6 (Tossiyskaya) | 2,083 | 2,128 | 1,852 | 2,532 | 1,918 |

Catalase activity, ml O _2_ /g min

| Cultivars | **A (Control)** | **B** | **C** | **D** | **E** |
| --- | --- | --- | --- | --- | --- |
| 1 (Coreggiolo) | 66.7 | 5.5 | 11.9 | 4.5 | 76.9 |
| 2 (Leccino) | 68.0 | 19.5 | 36.5 | 39.1 | 36.1 |
| 4 (Razzo) | 38.7 | 71.4 | 22.1 | 26.3 | 43.3 |
| 3 (Nikitskaya-2) | 33.8 | 25.5 | 33.1 | 20.4 | 16.1 |
| 5 (Tiflisskaya) | 62.9 | 34.0 | 37.4 | 54.4 | 42.1 |
| 6 (Tossiyskaya) | 28.9 | 11.0 | 19.5 | 11.3 | 23.4 |
