# Supplementary material for: Freeze-Dependent Physiological and Transcriptional Changes in Olea europaea L. Cultivars with Different Cold Resistances
Source: Int J Mol Sci. 2025 Apr 22;26(9):3934. doi: 10.3390/ijms26093934 (PMC12071819; doi:10.3390/ijms26093934)
Supplement: Supplementary file 1 [file ijms-26-03934-s001.zip › Supplementary/Supplementary File S6.docx]

**Transcript list for qPCR validation:**

| **Transcript** | **Pearson correlation coefficient** | **Forward primer** | **Reverse primer** | **Fragment length, bp** |
| --- | --- | --- | --- | --- |
| Oeu004464.1 | r^2^ < 1 | GCAATCACGAGGTCAACATG | GCGTAATCTGCTGCATCGAC | 170 |
| Oeu001505.1 | r^2^ < 1 | GATACGAGAGCATTTGGGTC | TCGGGCCTCTCTGTGAAAAG | 163 |
| Oeu013164.1 | r^2^ < 1 | TACTCATAGACATACATAG | CCAAGCAAATATGGTGAAGC | 161 |
| Oeu013764.1 | r^2^ < 1 | GCGGAGAAACGTAGAAGCTG | CCACAAATGCTCTTGTGATC | 157 |
| Oeu023842.1 | r^2^ < 1 | AGCCAAGAATCCAAGCTTG | TCGGACCCAACTTCATCTCC | 177 |
| Oeu007422.1 | r^2^ < 1 | GGTTAGGATCATACGAGACG | ATCTGCGGCGGAGACAATTC | 144 |
| Oeu045215.1 | r^2^ < 1 | AAATACTCAGTCGTGCCGCC | GAGGAGGTCTTTGACGGAAG | 149 |
| Oeu048245.1 | r^2^ < 1 | TTTCGTTCAGCACTTCGCCG | GCAAATGCCCGGAGAATATC | 165 |
| Oeu057291.1 | r^2^ < 1 | TTTAAGCAACGTACTCCCAG | CGGAGTGCGAGTTGTTTTGC | 158 |
| Oeu031399.1 | r^2^ < 1 | TGGTCGAGGCCCATGTATTG | TTGGCCAACCATTGATCCAC | 165 |
| Oeu046137.1 | r^2^ < 1 | GAGTTTCCTCTCACGAATGG | GGAATTGGAGTGACTGTGTTG | 211 |
| Oeu045100.1 | r^2^ < 1 | CTGAAAGACATACTTCCGGC | TCCCTTCATCGTGCTTAAGC | 157 |
| GAPDH | House-keeping | TTCTCTCCTTGGATCTGCAC | AACCACGGGTAGCAGTCATC | 185 |


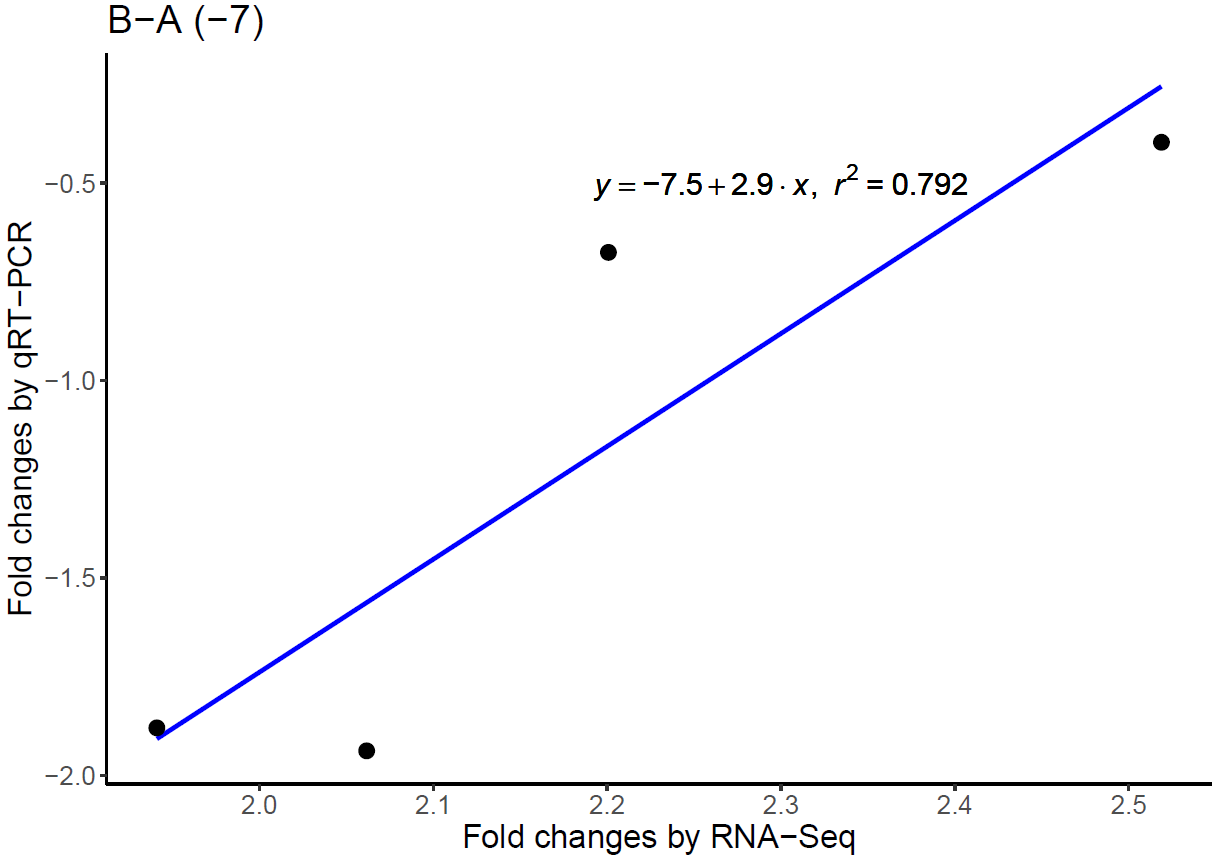


**Supplementary Figure 6.1.** Results of separate validation for *Oeu046137.1* and *Oeu045100* for B vs A comparison at –7°C. Each dot represents each transcript in cold-tolerant and heat-loving cultivars separately.


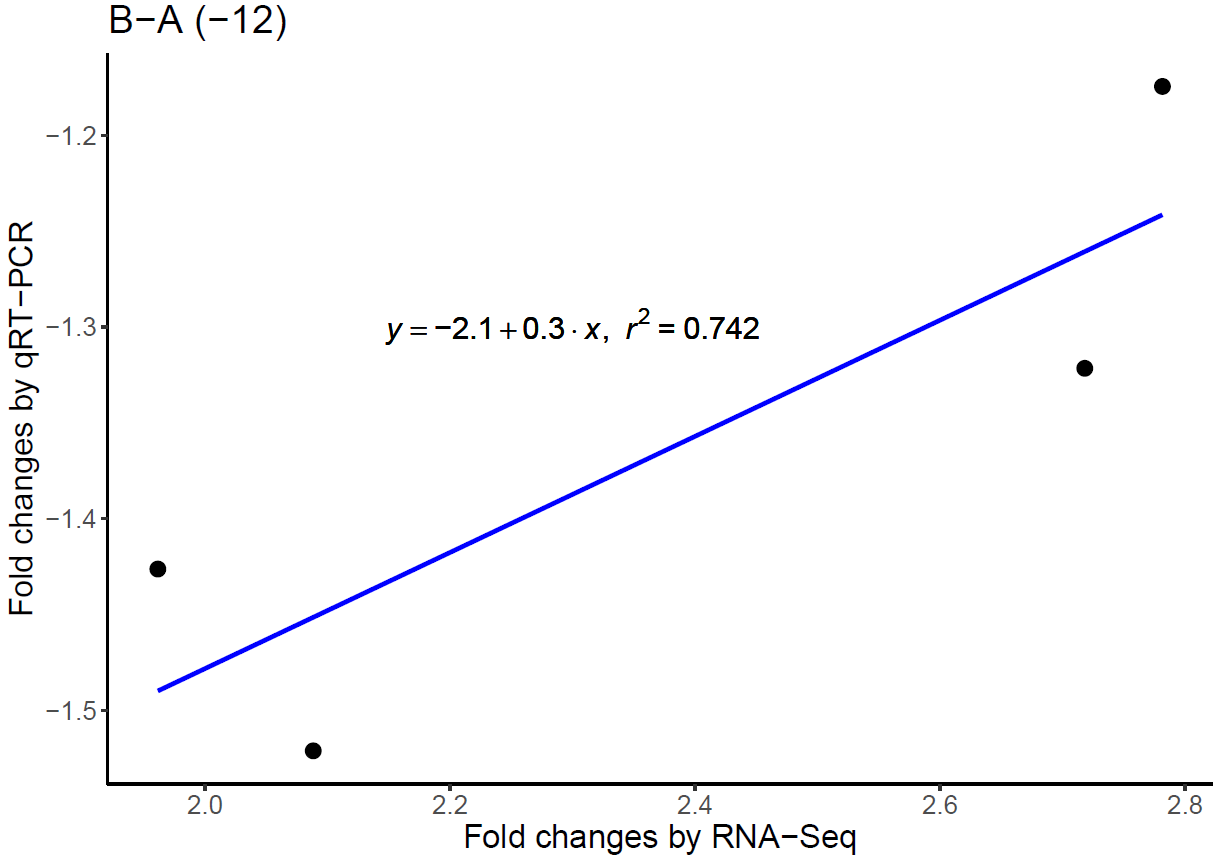


**Supplementary Figure 6.1.** Results of separate validation for *Oeu046137.1* and *Oeu045100* for B vs A comparison at –12°C. Each dot represents each transcript in cold-tolerant and heat-loving cultivars separately.
